# Exploring the Spatial Relationships Between Real and Virtual Experiences: What Transfers and What Doesn’t

**DOI:** 10.3389/frvir.2020.572122

**Published:** 2020-10-08

**Authors:** Gregory D. Clemenson, Lulian Wang, Zeqian Mao, Shauna M. Stark, Craig E. L. Stark

**Affiliations:** Department of Neurobiology and Behavior, University of California, Irvine, Irvine, CA, United States

**Keywords:** transfer, real-world navigation, virtual reality, spatial cognition, virtual environments (VE), real-environments

## Abstract

Virtual environments are commonly used to assess spatial cognition in humans. For the past few decades, researchers have used virtual environments to investigate how people navigate, learn, and remember their surrounding environment. In combination with tools such as electroencephalogram, neuroimaging, and electrophysiology, these virtual environments have proven invaluable in their ability to help elucidate the underlying neural mechanisms of spatial learning and memory in humans. However, a critical assumption that is made whenever using virtual experiences is that the spatial abilities used in the navigation of these virtual environments accurately represents the spatial abilities used in the real-world. The aim of the current study is to investigate the spatial relationships between real and virtual environments to better understand how well the virtual experiences parallel the same experiences in the real-world. Here, we performed three independent experiments to examine whether spatial information about object location, environment layout, and navigation strategy transfers between parallel real-world and virtual-world experiences. We show that while general spatial information does transfer between real and virtual environments, there are several limitations of the virtual experience. Compared to the real-world, the use of information in the virtual-world is less flexible, especially when testing spatial memory from a novel location, and the way in which we navigate these experiences are different as the perceptual and proprioceptive feedback gained from the real-world experience can influence navigation strategy.

## INTRODUCTION

Spatial navigation is a fundamental behavior that is shared amongst humans and non-human animals alike. The ability to navigate, learn, and remember our surrounding environment is critical for everyday life and requires the coordination of numerous perceptual and sensory processes of both self-motion and environmental cues (Lester et al., 2017). These processes are further supported by a network of brain regions, including the hippocampus, retrosplenial cortex, striatum, and entorhinal cortex in order to navigate and create successful representations of space (O’Keefe and Nadel, 1978; Ekstrom et al., 2003; Hartley et al., 2003; Marchette et al., 2011; Chrastil et al., 2015; Moser et al., 2015; Huffman and Ekstrom, 2019). While initial studies of these underlying spatial neural networks stemmed from *in-vivo* recordings of awake behaving non-human animals in a real-world environment (Tolman, 1948; O’Keefe and Nadel, 1978; Moser et al., 2015), studies in both humans and non-human animals have demonstrated that similar neural networks are active even during the navigation and exploration of virtual environments (Ekstrom et al., 2003; Harvey et al., 2009; Woollett et al., 2009; Jacobs et al., 2013; Schmidt-Hieber and Häusser, 2013; Huffman and Ekstrom, 2019). These data suggest that within the spatial domain, the same neural architecture is involved in processing and offers potential for transfer between real and virtual spatial experiences. Here, within the spatial domain, we investigate the transfer of spatial knowledge between real and virtual environments.

Transfer between real and virtual experiences have been previously observed across various situations. For example, several studies have shown that virtual experiences are valid tools for assessing human evacuation behaviors in response to social, stressful, and potentially dangerous situations. When placed in a crowded and stressful virtual experience, participants are influenced by virtual bystanders, behaving in ways that parallel a similar real world situation (Kinateder and William, 2016). Studies have used these virtual crowd simulators to better understand evacuation behavior of crowds in highly stressful situations and strategies to manage these risky circumstances (Moussaïd et al., 2016; Zhao et al., 2020). Similar virtual experiences have even been used to probe navigation decisions of individuals when faced with a choice between following the crowd or a map (Zhao et al., 2020).

Spatial processing in humans is commonly assessed using virtual environments via traditional desktop computers, virtual reality head mounted displays, virtual projection rooms, video games, and smart phone applications (Chance et al., 1998; Richardson et al., 1999; Maguire et al., 2000; Ekstrom et al., 2003; Jacobs et al., 2013; Chrastil et al., 2015; Kimura et al., 2017; Clemenson et al., 2019; Coutrot et al., 2019; Diersch and Wolbers, 2019; Patai et al., 2019; Hejtmanek et al., 2020). Occasionally these methods are paired with neuroimaging, electroencephalogram (EEG), and in some cases, electrophysiology, to investigate their underlying neural mechanisms. While many of these behavioral studies of navigation within virtual environments parallel the work performed in animals, it is not clear how accurately these methods of spatial navigation in humans reflect spatial abilities in the real world, as the cues for self-motion, body orientation, distance, and speed, which are important for spatial navigation, are limited in the virtual experience (Taube et al., 1990; Chance et al., 1998; Richardson et al., 1999; McNaughton et al., 2006; Kraus et al., 2015; Kropff et al., 2015; Shine et al., 2016).

The use of virtual experiences to evaluate spatial memory in humans suggests a close relationship between how the brain perceives both real and virtual experiences. The hippocampus plays a critical role in spatial learning and memory and contains a network of neurons dedicated to encoding space (Tolman, 1948; O’Keefe and Dostrovsky, 1971; O’Keefe and Nadel, 1978; Moser et al., 2015). Similar neural networks within the hippocampus are also active during spatial tasks within a virtual environment (Ekstrom et al., 2003; Woollett and Maguire, 2011; Jacobs et al., 2013; Huffman and Ekstrom, 2019). Much like the benefits non-human animals receive from the spatial exploration of a real world environment (Freund et al., 2013; Clemenson et al., 2018), the exploration of virtual environments found within video games can lead to improvements in hippocampal memory (Clemenson and Stark, 2015; Clemenson et al., 2019, 2020). In non-human animals, aging is closely associated with a decline in spatial memory (Bizon and Gallagher, 2003; Drapeau et al., 2003; van Praag et al., 2005) and in humans this decline, as measured using virtual navigation (Konishi and Bohbot, 2013; Kolarik et al., 2016; Lester et al., 2017), can even predict the conversion from mild cognitive impairment to Alzheimer’s disease (Cushman et al., 2008; Laczó et al., 2010).

In addition to spatial memory, spatial navigation is commonly assessed with the use of virtual environments. Representations of space are formed through navigation (Tolman, 1948) and are commonly separated into two types of navigation strategies: allocentric and egocentric. Allocentric navigation describes how cues within the environment relate to one another (a map). Egocentric navigation describes how cues within the environment relate to the individual (a set of directions). Here, we utilize the T-maze task to dissociate between place learning and route learning (Packard and McGaugh, 1996; Tomás Pereira et al., 2015). Importantly, while these two forms of learning are often directly compared to allocentric and egocentric navigation, place learning and route learning are more straight forward and do not require the same spatial reference frames as true allocentric and egocentric navigation (Wolbers and Wiener, 2014). While both strategies promote successful navigation, early non-human animal studies suggested that they were supported by independent networks. The hippocampus and surrounding medial temporal lobe areas of the brain have long been implicated in the formation of allocentric representations of space (Tolman, 1948; O’Keefe and Dostrovsky, 1971; O’Keefe and Nadel, 1978), whereas the caudate nucleus and other striatal regions are necessary for the formation of egocentric representations of space (Cook and Kesner, 1988; Kesner et al., 1993; Packard and McGaugh, 1996). Recent works, however, suggests that the strict dissociation between hippocampal and striatal spatial networks are not as clear as we once thought (Wolbers and Wiener, 2014; Goodroe et al., 2018). In humans, similar regions are active during the navigation of virtual environments (Iaria et al., 2003; Bohbot et al., 2007; Huffman and Ekstrom, 2019), suggesting real potential for transfer of spatial knowledge between real and virtual environments.

Despite these commonalities in neural substrates, a key difference between real and virtual experiences is the amount of perceptual and proprioceptive feedback we receive from the experience. Several studies have explored the impact of vestibular and proprioceptive inputs on navigation and while it is evident that spatial information can be learned from virtual experiences with limited inputs, there are clear advantages to the real world experience (Chance et al., 1998; Richardson et al., 1999; Hejtmanek et al., 2020). Importantly, a recent study showed that when learning a large-scale virtual environment, the underlying neural networks involved in the retrieval of that spatial knowledge was not influenced by the number of body-based cues (Huffman and Ekstrom, 2019). Regardless of whether spatial knowledge was acquired through simple visual inputs (computer screen and a joystick to move) or with more enriched body movements (treadmill and virtual reality headset), the neural networks underlying the retrieval of that spatial knowledge were similar.

Here, we recreated real-world locations within a virtual environment to directly address whether spatial information transferred between real and virtual environments. In Experiments 1 and 2, we found that while general information about both object location and maze layout transferred between experiences, there were significant benefits of the real-world experience, especially when using the spatial information from a novel location. In Experiment 3, we demonstrate that the way we experience virtual environments (such as the use of a virtual reality headset) can have a dramatic influence on navigation strategy. Together, these three experiments explore the spatial relationship between real and virtual experiences and begin to address how well the virtual experience parallels the real experience.

## EXPERIMENT 1: OBJECT LOCATION TASK (OLT)

The goal of the OLT task was to investigate, in a simple way, whether spatial knowledge transferred between real and virtual environments. We designed a spatial memory task in which participants learned the spatial locations of objects hidden within an environment through a pre-exposure. After the pre-exposure, participants were tested on the spatial locations of the objects in opposing environments (pre-exposed in the real and tested in the virtual environment or pre-exposed in the virtual and tested in the real environment). Then, we compared their performance with both negative controls (no pre-exposure and tested only in one environment) and positive controls (pre-exposed and tested in the same environments) to determine if spatial information transferred between real and virtual environments.

### MATERIALS AND METHODS

#### Participants

**FIGURE 1 |:**
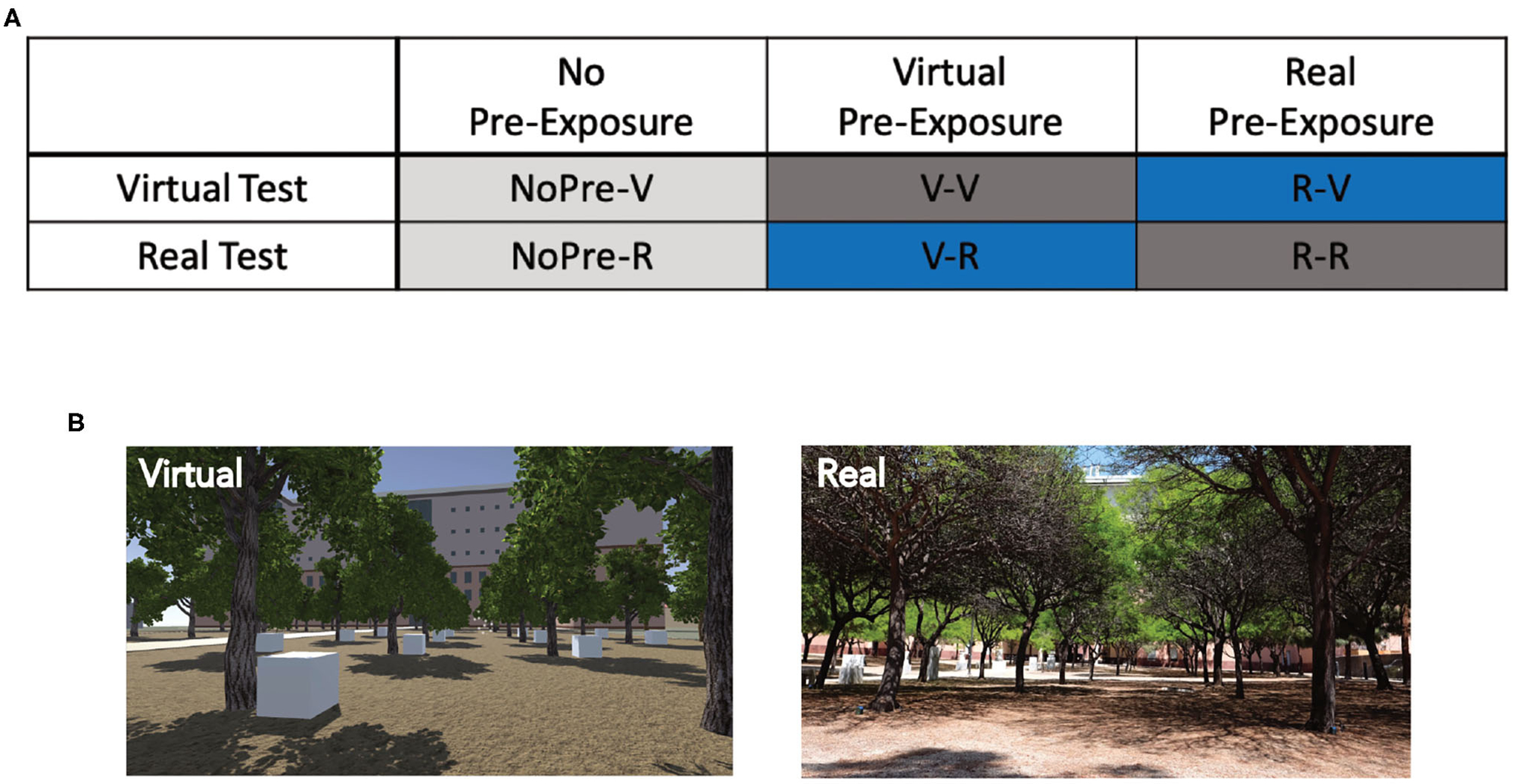
Experimental design and groups for Experiment 1 (OLT). **(A)** The six different groups used in Experiment 1 based on the pre-exposure and testing environments. Negative controls, NoPre-V and NoPre-R; positive controls, V-V and R-R; and experimental groups, V-R and R-V. **(B)** Example images of the real and virtual versions of the OLT.

In total, 77 participants (41 female, 36 male; Mean age: 20.83 years, SD: 2.78) were recruited through the Sona Systems experimental management system at the University of California at Irvine, which organizes the participation of students in science experiments for course credit. Participants were randomly placed into one of six groups (Figure 1A; see below for detailed group descriptions): No pre-exposure and virtual test (NoPre-V; 6 female, 6 male), no pre-exposure and real test (NoPre-R; 6 female, 6 male), virtual pre-exposure and virtual test (V-V; 7 female, 6 male), real pre-exposure and real test (R-R; 11 female, 6 male), virtual pre-exposure and real test (V-R; 5 female, 6 male), and real pre-exposure and virtual test (R-V; 6 female, 6 male). All participants signed consent forms approved and conducted in compliance with the Institutional Review Board of the University of California at Irvine.

#### Object Location Task (OLT)

**FIGURE 2 |:**
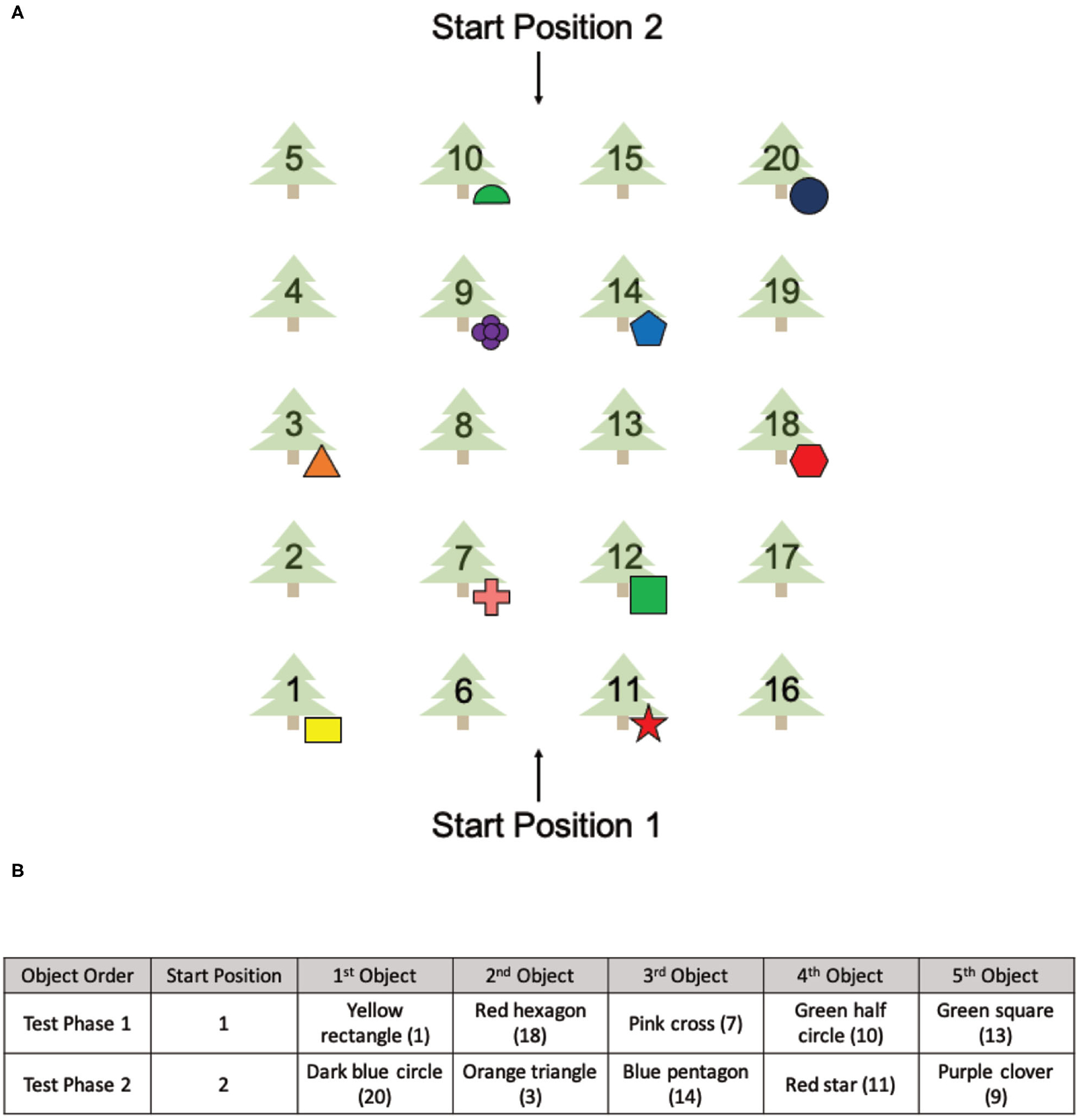
The spatial layout of the OLT and the environmental exposures of all six groups. **(A)** The spatial layout of the items used in the OLT with two different starting positions. **(B)** The order of the objects found in Test Phase 1 and Test Phase 2.

The OLT consisted of a spatial memory task designed for use in both real and virtual environments (Figure 1B). The OLT consisted of one pre-exposure (learning) phase and two test phases, in which participants were tested on their knowledge of 10 hidden objects amongst 20 possible locations. In both versions of the OLT, the environment arena consisted of a grove of 20 trees arranged in a 4 × 5 grid pattern with two starting positions at opposing sides of the arena (Figure 2A). A container was placed at the base of each of the 20 trees (details about the real and virtual versions of the OLT are described below). Ten of the containers contained different colored geometric shapes and the remaining 10 containers were empty.

The Pre-Exposure Phase involved a 5-min free exploration of either the virtual environment or the real environment. From Start Position 1 (Figure 2A), participants were instructed that there were 10 objects (colored geometric shapes) hidden amongst 20 possible locations and their goal was to find and remember the spatial locations of each object.

During both Test Phase 1 and Test Phase 2, participants were asked to find and retrieve five objects in either the virtual environment or the real environment (Figure 2A), one at a time starting from Start Position 1 (Test Phase 1) or Start Position 2 (Test Phase 2). The order of the objects was the same for both test phases and all participants (Figure 2B). From each start position, participants were instructed to find a single object and once found, return to the same start position.

Importantly, the five baited containers used in each test phase were strategically arranged to be isomorphic, ensuring that the spatial layout was the same from both start locations (Figure 2A). Thus, all objects used in Test Phase 1 had a counterpart object in Test Phase 2 that existed in the same spatial location with respect to the associated start position. The counterpart object of the yellow rectangle (tree 1) was the dark blue circle (tree 20), the counterpart object of the red hexagon (tree 18) was the orange triangle (tree 3), the counterpart object of the pink cross (tree 7) was the blue pentagon (tree 14), the counterpart object of the green half circle (tree 10) was the red star (tree 11) and the counterpart object of the green square (tree 12) was the purple clover (tree 9). This layout allowed us to probe navigational strategies employed during retrieval.

#### Real-World Object Location Task (Real-OLT)

The Real-OLT was performed in a grove of 20 trees arranged in a 4 × 5 grid (100 × 75 feet), located behind the Science Library at the University of California Irvine (Figure 1B). Twenty, 6-inch green plastic flowerpots were placed upside down behind all 20 trees, in plain sight. Underneath 10 of the flowerpots were 10 wooden blocks of various colors and shapes (all different), attached to the inside of the flowerpots using Velcro, along with a wireless tag (www.wirelesstag.net; CaoGadgets LLC, 2010) to record when the pot was turned over. The number of errors was recorded by the wireless tags inside the pots, as well as by two independent experimenters. An error was only recorded if the participant picked up the pot and turned it over to look at the object inside. Importantly, during Test Phase 2, when participants switched start locations, participants followed the experimenter around the outside of the arena to the second start location, emphasizing the shift in spatial layout.

#### Virtual-World Object Location Task (Virtual-OLT)

The Virtual-OLT was created using a combination of Unity (www.unity.com; Technologies Unity, 2005) and SketchUp (www.sketchup.com; Trimble, 2000), to recreate the Real-OLT scene in a virtual world, including all visible buildings and structures. Once the scene was created, Unity was used to develop, run, and collect data for the behavioral task. We intentionally designed the Virtual-OLT to look and feel like the Real-OLT, using Google Maps (www.google.com/maps; Google LLC, 2005) to ensure that the spatial layouts and distances matched the Real-OLT as best as possible (Figure 1B). Twenty virtual trees were placed in the same spatial layout as the Real-OLT and 20 white boxes were placed at the base of every tree with 10 of the white boxes containing a colored, geometric shape (Figure 2A). Importantly, the color, shape, and spatial location of the objects used in the Real-OLT and Virtual-OLT were the same. Errors were recorded by the Virtual-OLT program.

The Virtual-OLT was performed on an iMac, using the mouse and keyboard. Prior to starting the task, participants were given simple instructions on how to navigate the arena using the W, A, S, and D keys. Clicking the white box with the mouse revealed the object underneath. As some participants had difficulty using the keyboard and mouse to navigate, white boxes were used in place of green flowerpots in the Virtual-OLT. This made it easier for all participants to find the containers since we were testing participants’ spatial memory for the objects, not their ability to find the containers. Importantly, during Test Phase 2 when participants switched start locations, participants were teleported to the second start location and explicitly told that they would be starting from the opposite side of the maze.

#### Experimental Groups

Overall, there were three pre-exposure conditions (no pre-exposure, virtual pre-exposure, and real pre-exposure) and two testing conditions (virtual test and real test), for a total of six groups (Figure 1A). In every case, the test condition represented the condition (real or virtual) that participants were tested in for both Test Phase 1 and Test Phase 2. The no pre-exposure conditions (NoPre-V and NoPre-R) contained negative controls who were not given a Pre-Exposure Phase but instead, went straight to Test Phase 1 and Test Phase 2. This condition allowed us to quantify the probability of participants finding the objects in either environment (virtual or real) without any prior knowledge of the environment. We expected these no pre-exposure groups to make the most errors of all groups. The groups that were pre-exposed and tested in the same environment (V-V and R-R) represented positive controls, exposed to the ideal pairing of pre-exposure environment and test environment. We expected these groups to perform the best, making the least number of errors. The groups that were pre-exposed and tested in alternate environments (V-R and R-V) composed the experimental conditions, exploring the transfer of knowledge between real and virtual environments.

#### Statistical Analyses

Statistical analyses were performed using Prism 7 (GraphPad Prism). Bayesian analyses and effect sizes were performed using JASP (www.jasp-stats.org; Anon, 2019). Specific statistical tests used are reported with the results. A statistical *p*-value of 0.05 was used for all analyses.

### RESULTS—EXPERIMENT 1

#### Is There Evidence of Object-Location Learning in Both Real and Virtual Environments?

**FIGURE 3 |:**
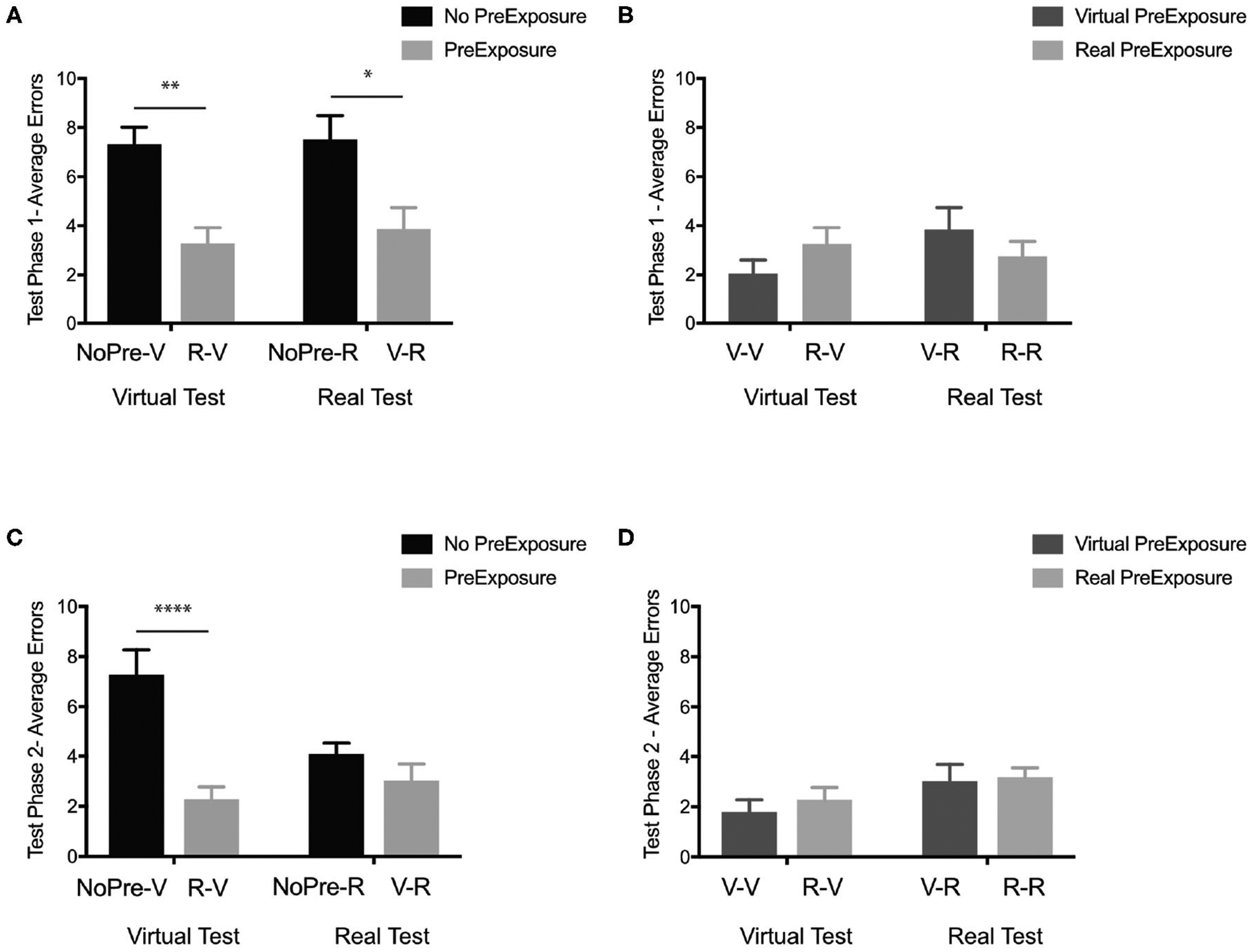
In the OLT, general spatial information transferred between real and virtual environments. **(A)** Test Phase 1 performance (average errors) of the negative controls without pre-exposure (black; NoPre-V and NoPre-R) and experimental groups exposed to incongruent experiences (light gray; V-R and R-V). **(B)** Test Phase 1 performance (average errors) of the positive controls exposed to congruent experiences (dark gray; V-V and R-R) and experimental groups exposed to incongruent experiences (light gray; V-R and R-V). **(C)** Test Phase 2 performance (average errors) of the negative controls without pre-exposure (black; NoPre-V and NoPre-R) and experimental groups exposed to incongruent experiences (light gray; V-R and R-V). **(D)** Test Phase 2 performance (average errors) of the positive controls exposed to congruent experiences (dark gray; V-V and R-R) and experimental groups exposed to incongruent experiences (light gray; V-R and R-V). All data are presented as mean ± SEM, *p < 0.05, **p < 0.01, ****p < 0.0001.

Our first question was whether any spatial information was learned in the experimental groups (V-R and R-V) even with incongruent pre-exposure and testing conditions. Using the performance (average number of errors) at Test Phase 1 of both the negative control groups (NoPre-V and NoPre-R) and the experimental groups (V-R and R-V), we performed a 2 × 2 ANOVA across pre-exposure (with and without pre-exposure) and two test conditions (virtual and real) to determine whether a pre-exposure of the opposing experience could promote learning. In the initial test phase (Test Phase 1), we found a significant main effect of pre-exposure, Figure 3A; *F*_(1, 43)_ = 21.67, *p* < 0.0001, *η*^2^ = 0.334, but no main effect of test condition, *F*_(1, 43)_ = 0.22, *p* = 0.63, *η*^2^ = 0.003, or interaction, *F*_(1, 43)_ = 0.05, *p* = 0.81, *η*^2^ = 0.001. *Post-hoc* analyses (Sidak’s correction for multiple comparisons) revealed that there was no difference between the negative controls (NoPre-V and NoPre-R), regardless of testing environment, and no difference between experimental conditions (V-R and R-V), regardless of testing environment. These data suggest that at the most basic level, the real and virtual conditions were similarly challenging, and spatial information transferred between real and virtual experiences.

#### What Is the Extent of Spatial Information Transfer Between Congruent (V-V and R-R) and Incongruent Experiences (V-R and R-V) of the OLT?

Next, we examined the extent of the transfer between the virtual and real experiences of the OLT. Using the performance at Test Phase 1 of both the positive control groups (V-V and R-R) and the experimental groups (V-R and R-V), we performed a 2 × 2 ANOVA across pre-exposure conditions (virtual and real) and test conditions (virtual and real). In the initial test phase (Test Phase 1), we found no main effect of pre-exposure condition (Figure 3B), *F*_(1, 49)_ = 0.007, *p* = 0.93, *η*^2^ = 0.00, or test condition, *F*_(1, 49)_ = 0.89, *p* = 0.35, *η*^2^ = 0.02, with a slight trend toward an interaction, *F*_(1, 49)_ = 2.78, *p* = 0.10, *η*^2^ = 0.05. In order to better understand these null effects, we ran a Bayesian analysis and found that the null model was the best predictor of the data, with all other models being less likely (test condition, BF_10_ = 0.36, pre-exposure condition, BF_10_ = 0.28, test condition + pre-exposure + interaction, BF_10_ = 0.10, and interaction, BF_10_ = 0.10). These data suggest that the spatial information that transferred between incongruent experiences (V-R and R-V) transferred similarly to congruent experiences (V-V and R-R).

#### How Flexible Is the Transfer of Information When Starting From a Novel Location?

During Test Phase 2, we determined how flexibly participants could use the information acquired in the Pre-Exposure Phase (if present) and Test Phase 1 by starting them from a novel location (Start Position 2). Using the performance (average number of errors) at Test Phase 2 of both the negative control groups (NoPre-V and NoPre-R) and the experimental groups (V-R and R-V), a 2 × 2 ANOVA across pre-exposure (with and without pre-exposure) and two test conditions (virtual and real) revealed a significant main effect of pre-exposure (Figure 3C), *F*_(1, 43)_ = 18.57, *p* < 0.0001, *η*^2^ = 0.26, a trend toward a main effect of test condition, *F*_(1, 43)_ = 3.012, *p* = 0.08, *η*^2^ = 0.04, and a significant interaction, *F*_(1, 43)_ = 7.83, *p* < 0.01, *η*^2^ = 0.11. *Post-hoc* analyses (Sidak’s correction for multiple comparisons) revealed that the NoPre-V group (M = 7.27, SD = 3.47) performed significantly worse than the NoPre-R group (M = 4.07, SD = 1.65), the R-V group (M = 2.25, SD = 1.82), and the V-R group (M = 3.00, SD = 2.28). These data suggest that when tested from a novel location, there is a difference between real and virtual experiences. Comparing performance on Test Phase 1 and Test Phase 2 for the NoPre-V and NoPre-R groups in a 2 × 2 ANOVA, we found a significant interaction [*F*_(1, 22)_ = 4.74, *p* = 0.04, *η*^2^ = 0.08], a significant main effect of test phase [*F*_(1, 22)_ = 4.92, *p* = 0.04, *η*^2^ = 0.08), and no significant main effect of group [*F*_(1, 22)_ = 2.98, *p* = 0.09, *η*^2^ = 0.12]. A *post-hoc* analysis revealed a significant difference between Test Phase 1 and Test Phase 2 for the NoPre-R group (*p* = 0.01) but not the NoPre-V group (*p* = 0.99). These data suggest that the NoPre-R group was able to learn from the prior real-world experience (Test Phase 1) leading to an improvement in performance in Test Phase 2, whereas the NoPre-V group did not learn from the prior virtual world experience (Test Phase 1).

#### Can Spatial Information Learned in the Virtual Experience Be Used Flexibly With an Additional Exposure?

While the NoPre-R group was able to flexibly learn spatial information from a single exposure (Test Phase 1) to the test environment, the NoPre-V group was not. To determine whether spatial information learned in the virtual experience could be used flexibly with an additional pre-exposure, we analyzed the V-V group. While the NoPre-V group received a single pre-exposure (Test Phase 1) to the environment prior to Test Phase 2, the V-V group received two exposures (Pre-Exposure Phase and Test Phase 1) to the environment prior to Test Phase 2. Using the performance (average number of errors) at Test Phase 2 of both the positive control groups (V-V and R-R) and the experimental groups (V-R and R-V), a 2 × 2 ANOVA across pre-exposure condition (virtual and real) and test condition (virtual and real) revealed a main effect of test condition (Figure 3D), *F*_(1, 49)_ = 4.20, *p* = 0.04, *η*^2^ = 0.08, but not pre-exposure condition, *F*_(1, 49)_ = 0.37, *p* = 0.54, *η*^2^ = 0.01, or interaction, *F*_(1, 49)_ = 0.09, *p* = 0.75, *η*^2^ = 0.002. *Post-hoc* analyses (Sidak’s correction for multiple comparisons) revealed no differences between any groups. These data suggest that while we previously did not find any improvement in the negative control group exposed to the Virtual-OLT (NoPre-V), flexible learning can occur with an additional pre-exposure to the Virtual-OLT (V-V).

## EXPERIMENT 2: OBJECT LOCATION MAZE (OLM)

The goal of the OLM was to not only explore the transfer of spatial information between real and virtual environments but also to investigate the type of spatial information that transfers. The biggest difference between the OLT from Experiment 1 and the OLM used here was that while both tasks required a spatial knowledge of the objects and their relative spatial locations within the environment, the OLM added a navigation component due to the presence of a physical maze. In addition, while the OLT contained 10 objects hidden amongst 20 possible locations, the OLM contained eight hidden objects amongst eight possible locations within the maze. Like the OLT, the OLM started with a pre-exposure phase followed by two test phases (starting in opposing locations).

### MATERIALS AND METHODS

#### Participants

**FIGURE 4 |:**
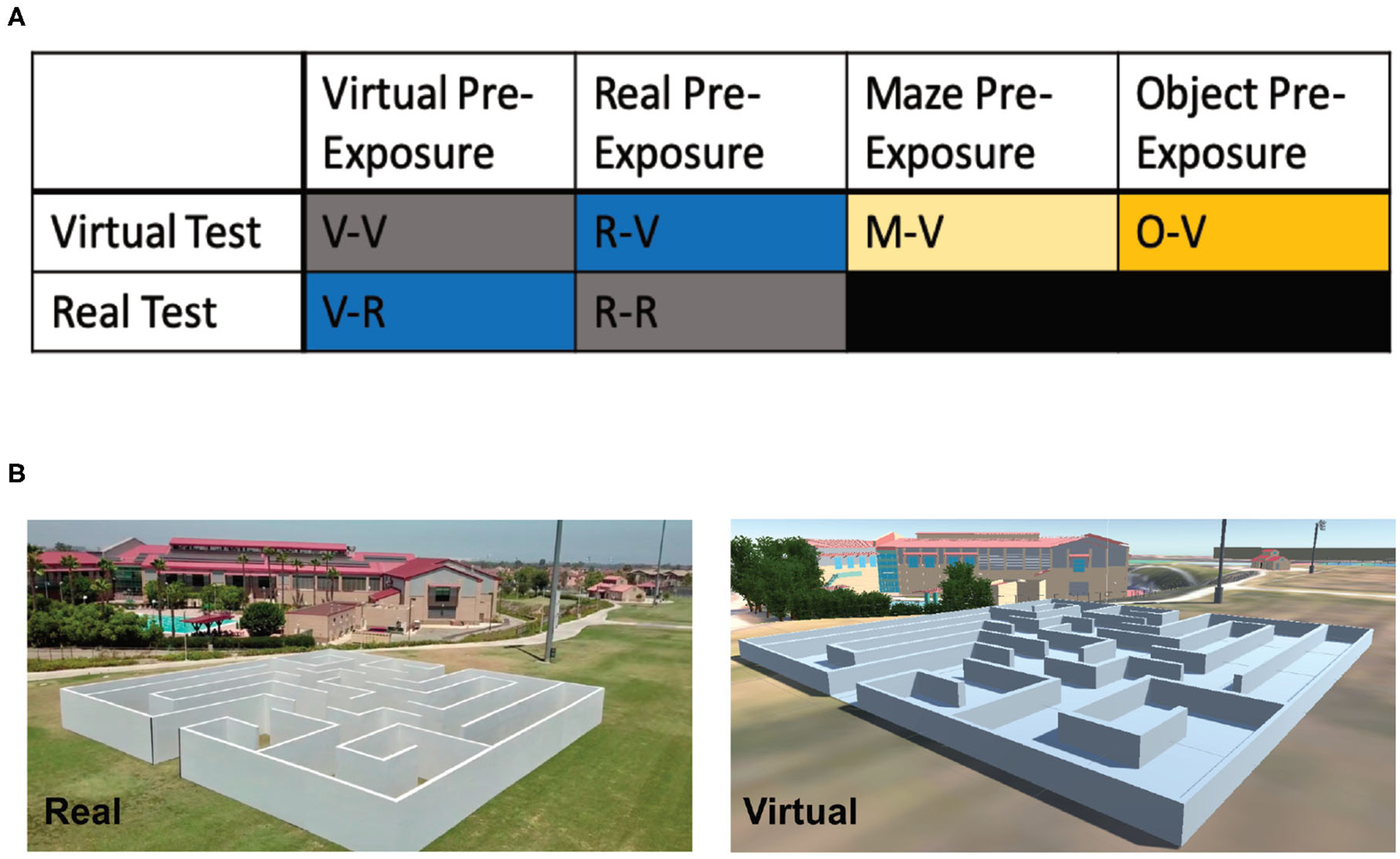
Experimental design and groups for Experiment 2 (OLM). **(A)** The six different groups used in Experiment 2 based on the pre-exposure and testing environments. **(B)** Example images of the real and virtual OLM.

In total, 81 participants (40 female, 41 male; Mean age: 22.72 years, SD: 5.71) were recruited through a combination of the UCI Sona Systems, an email blast to UCI students (ZOT Blast), and word of mouth. Participants were pseudo-randomly placed into one of six groups (Figure 4A; see below for detailed group descriptions): Virtual pre-exposure and virtual test (V-V; 6 female, 9 male), real pre-exposure and real test (R-R; 5 female, 6 male), virtual pre-exposure and real test (V-R; 5 female, 6 male), real pre-exposure and virtual test (R-V; 7 female, 7 male), maze pre-exposure and virtual test (M-V; 8 female, 6 male), and object pre-exposure (O-V; 9 female, 6 male). All participants signed consent forms approved and conducted in compliance with the Institutional Review Board of the University of California at Irvine.

#### Object Location Maze (OLM)

**FIGURE 5 |:**
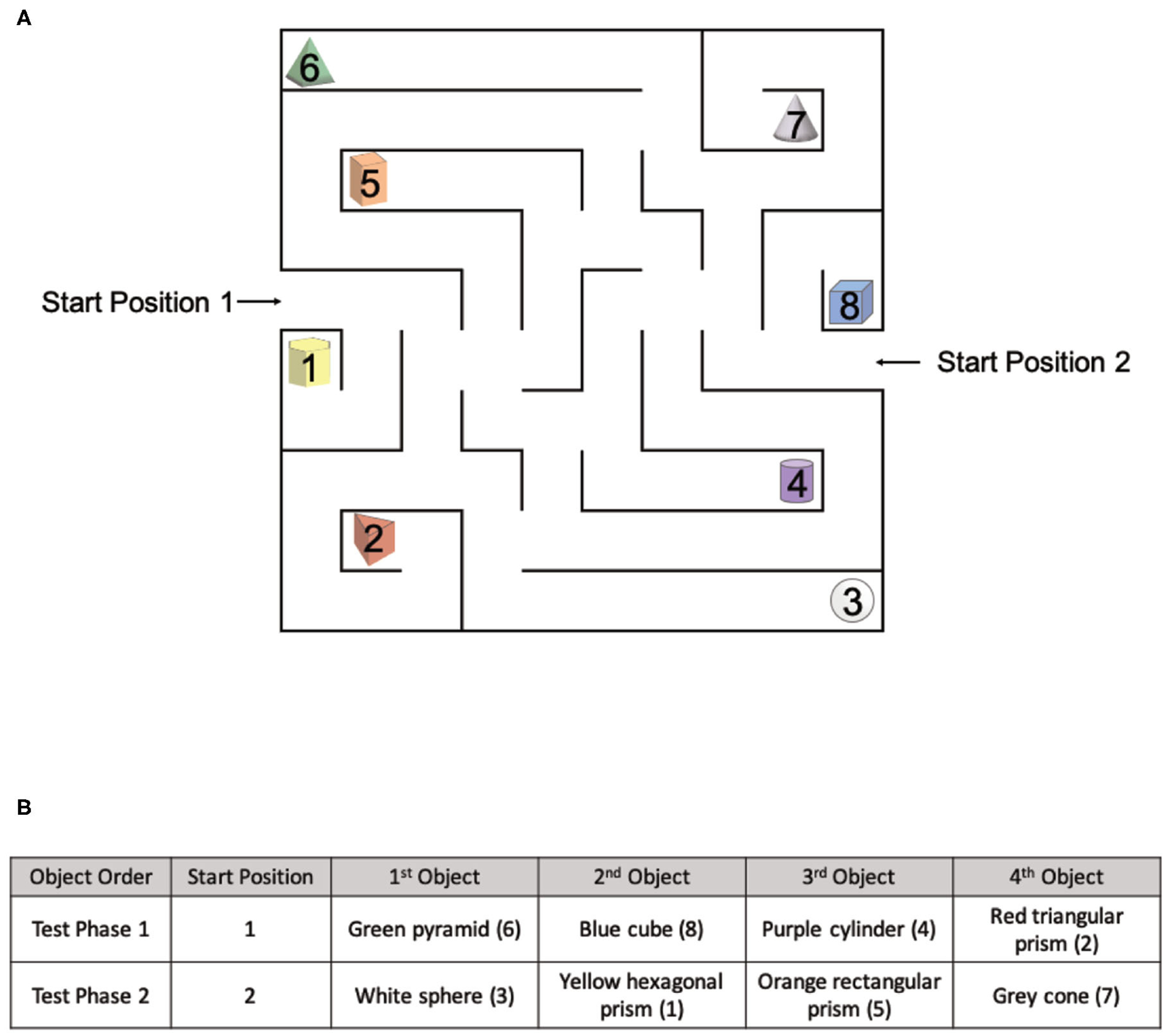
The spatial layout of the OLM and environmental exposures for each group. **(A)** The spatial layout of the maze and objects used in the OLM with two different starting positions. **(B)** The object order for Test Phase 1 and Test Phase 2.

The OLM consists of a spatial memory and navigation task designed for use in both real and virtual environments (Figure 4B). Similar to the OLT, the OLM consisted of one pre-exposure phase and two test phases. However, in the OLM participants navigated a maze to find eight hidden objects amongst eight possible locations. In both versions of the OLM, the environment arena consisted of a maze (Figure 5A) with eight dead-ends, each containing a different colored geometric shape, and two start positions. Importantly, the maze was designed to be isomorphic from each of the two start locations, ensuring that the spatial layout was the same from both start positions, allowing us to probe navigation strategies.

The Pre-Exposure Phase involved a 5-min free exploration of either the virtual environment or the real environment. From Start Position 1 (Figure 5A), participants were instructed that there were eight objects (colored geometric shapes) hidden within the maze and their goal was to find and remember the spatial locations of each object.

During Test Phase 1, participants were asked to find and retrieve four objects in either the virtual environment or the real environment, one at a time starting from Start Position 1 (Figure 5A) The order of the objects was the same for all participants (Figure 5B). From Start Position 1, participants were instructed to find a single object and once found, return to Start Position 1, repeated for all four objects.

During Test Phase 2, unlike the OLT, participants were asked to find and retrieve the remaining four objects in a specific order before returning to Start Position 2 (Figure 5A). The order of the objects was the same for all participants (Figure 5B). From Start Position 2, participants were instructed to find all objects in the required order before returning to Start Position 2. Test Phase 2 occurred in the same environment as Test Phase 1.

Importantly, the four baited arms used in each test phase were strategically arranged to be isomorphic, ensuring that the spatial layout was the same from both start locations. All objects used in Test Phase 1 had a counterpart object in Test Phase 2 that existed in the same spatial location with respect to the start position (Figure 5A). The counterpart object of the green pyramid (#6) was the white sphere (#3), the counterpart object of the blue cube (#8) was the yellow hexagonal prism (#1), the counterpart object of the purple cylinder (#4) was the orange rectangular prism (#5), and the counterpart object of the red triangular prism (#2) was the gray cone (#7).

#### Real World Object Location Maze (Real-OLM)

The Real-OLM was a custom-designed maze located on the lawn of the Anteater Recreation Center at the University of California, Irvine. The 60′ × 60′ × 6^′^ maze was made of wood and built by Mind Field, a web television series produced by YouTube Premium, for the episode “Your Brain on Tech” (https://youtu.be/1RHsAUyFCAM) filmed at UC Irvine. Inside, the maze contained eight dead ends and two starting positions (Figure 4B; designed by Gregory D. Clemenson and Craig E. L. Stark). At each terminal end was a colored geometric shape (eight different shapes in total), made from Styrofoam and wrapped in colored construction paper (during filming for the episode, real objects like a super-sized rubber duck and a seahorse plush were used).

For the Pre-Exposure Phase and Test Phase 1, the experimenter stood at Start Position 1 (Figure 5A) for the entire duration and tracked the time of each participant using a stopwatch. Prior to Test Phase 2, the participants followed the experimenter around the outside of the maze to Start Position 2. There, participants were given a slip of paper that listed the four objects, in order, they were to find. For the entire experiment, a camera was set on a tripod above the maze to record all participants’ movements. Participants wore a small backpack with a flag attached so that they were visible at all times to the camera. An experimenter, blind to the groups, recorded errors from the video footage. An error in the Real-OLM was defined by whether the participant had line-of-sight to an incorrect object. For example, if the participant peeked down a corridor with their head, breaking the invisible plane and giving them line-of-sight to the incorrect object, it was recorded as an error.

#### Virtual World Object Location Maze (Virtual-OLM)

Similar to the Virtual-OLT, the virtual version of the OLM (Figure 4B) was created and validated using a combination of Unity (www.unity.com; Technologies Unity, 2005), SketchUp (www.sketchup.com; Trimble, 2000), and Google Maps (maps.google.com; Google LLC, 2005). The colored, geometric objects and their spatial locations were identical in both the real and virtual versions of the OLM.

The Virtual-OLM was performed inside an office of the Anteater Recreation Center, on a MacBook Air with an external mouse and keyboard. During the Pre-Exposure Phase, participants were placed at Start Position 1 (Figure 5A) and asked to visit and remember all eight objects located within the maze. During Test Phase 1 the computer displayed each of the four objects the participant were supposed to find. Each object appeared at the top of the screen and was displayed until the object was found. Prior to Test Phase 2, participants were teleported to Start Position 2 and explicitly told that they were teleporting to the opposite side of the maze. Similar to Test Phase 1, during Test Phase 2 the computer displayed each of the four objects the participant were supposed to find. Each object appeared at the top of the screen and was displayed until the object was found. If the participant broke the plane to enter a corridor that led to an incorrect object, an error was recorded. All errors were recorded by the program.

#### Experimental Groups

In Experiment 2, participants were divided into six groups (Figure 4A; V-V, R-R, V-R, R-V, M-V, and O-V). These groups were used for two separate comparisons. The first comparison was a replication of Experiment 1, investigating whether general spatial knowledge transferred between real and virtual environments, and used the V-V, R-R, V-R, R-V groups. The second comparison investigated the type of spatial information that transferred and use the V-V, RV, M-V (maze only pre-exposure), and the O-V (object only pre-exposure) groups.

The first comparison, similar to the OLT, was designed to compare six groups (NoPre-V, NoPre-R, V-V, R-R, V-R, and R-V) across pre-exposure condition (no pre-exposure, virtual pre-exposure, and real pre-exposure) and test condition (virtual test and real test). However, due to time constraints of the filming (8 h), we were only able to test three of the groups (V-R, R-V, R-R) on the Real-OLM when it was available. The V-V group was run the following week. As we were unable to run the NoPre-V and NoPre-R groups due to time constraints, we simulated these groups by combining the groups that were pre-exposed to the virtual (V-V and V-R) and real (R-R and R-V) environments. The scores for the no pre-exposure groups (NoPre-V and NoPre-R) were derived from the Pre-Exposure Phase of the groups pre-exposed to either the virtual (V-R and V-V) environment or the real (R-V and R-R) environment, by scoring their initial Pre-Exposure Phase as if they were trying to find particular objects in a test phase without having had the benefit of a pre-exposure. In the Pre-Exposure Phase, participants learned all object-location pairs with the goal of finding those objects again during a later test. Thus, using the Pre-Exposure Phase of the V-R and V-V group (virtual pre-exposure) or R-V and R-R group (real pre-exposure), we calculated the number of errors participants made before they found the four objects of Test Phase 1 (#6, #8, #4, #2). To validate this approach, we ran the NoPre-V group and compared it to the simulated NoPre-V group (using the Learning Phase of V-V and V-R groups) and found similar results. It should be noted that we were only able to simulate the performance of the NoPre-V and NoPre-R groups for Test Phase 1 as the conditions would not have allowed us to simulate the learning that would have occurred from Test Phase 1 to Test Phase 2.

The second comparison used the five groups (Figure 4A) tested in the virtual environment (NoPre-V, V-V, R-V, M-V, and O-V) to investigate whether information about either the maze itself (M-V: maze only pre-exposure) or object location (O-V: object only pre-exposure) transferred from the pre-exposure. Due to time constraints for use of the Real-OLM, this last comparison was only tested in the Virtual-OLM. During the Pre-Exposure Phase of the M-V group, the maze walls were presented but the objects were removed from the maze. During the Pre-Exposure Phase of the O-V group, the maze walls were removed but the objects appeared in the correct spatial locations.

### RESULTS—EXPERIMENT 2

#### Is There Evidence of Maze Learning in Both Real and Virtual Maze Environments?

**FIGURE 6 |:**
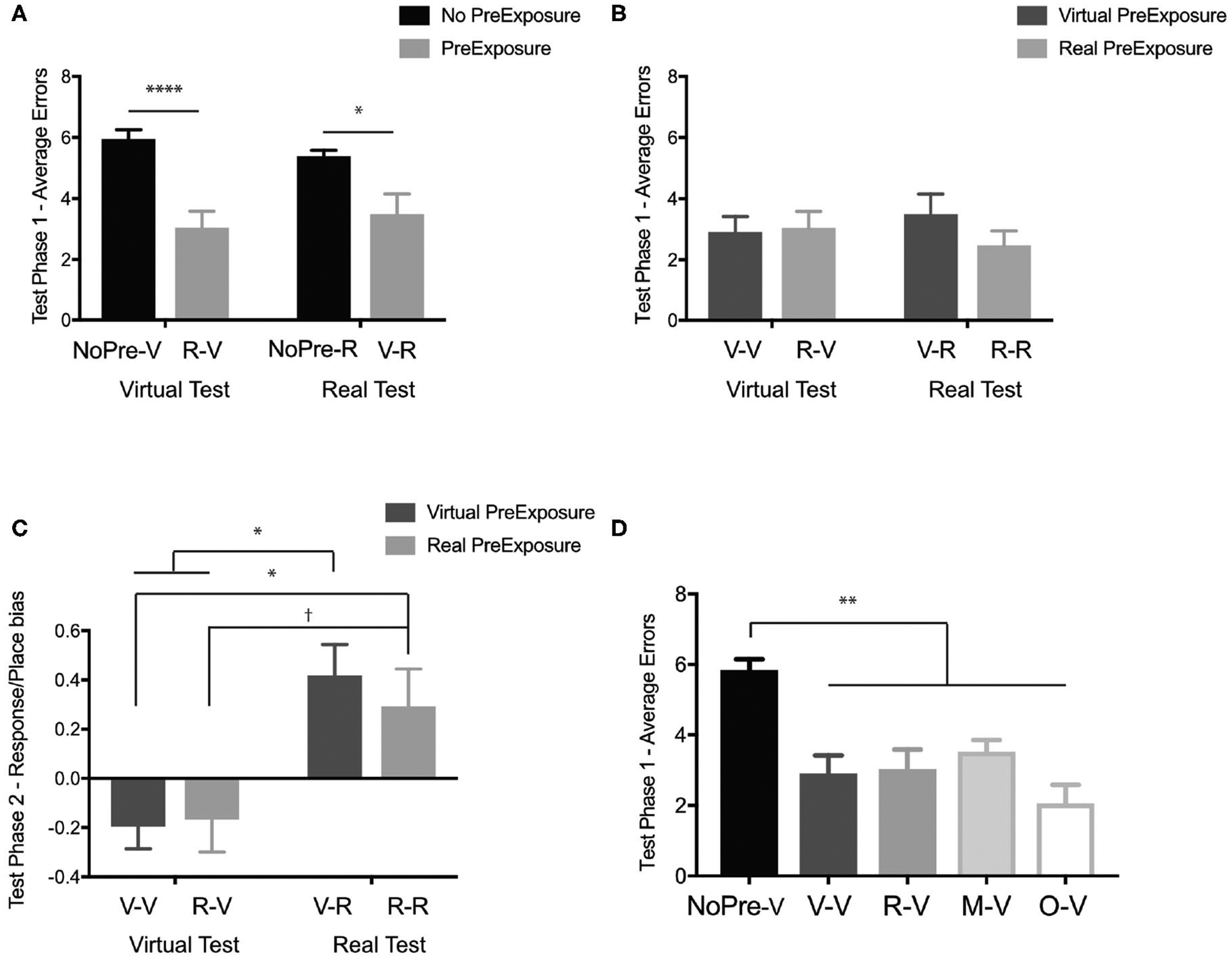
In the OLM, which required navigation, general spatial information transferred between real and virtual environments. **(A)** Test Phase 1 performance (average errors) of the negative controls without pre-exposure (black; NoPre-V and NoPre-R) and experimental groups exposed to incongruent experiences (light gray; V-R and R-V). **(B)** Test Phase 1 performance (average errors) of the positive controls exposed to congruent experiences (dark gray; V-V and R-R) and experimental groups exposed to incongruent experiences (light gray; V-R and R-V). **(C)** Navigation strategy biases (<0 is more response biased and >0 is more place biased) the groups tested in the virtual environment (V-V and R-V) and the groups tested in the real environment (V-R and R-R). **(D)** Test Phase 1 performance of all groups tested in the virtual environment (NoPre-V, V-V, and R-V) including the two groups pre-exposed to the maze-only condition (M-V) and object-only condition (O-V). All data are presented as mean ± SEM, *^†^p* < 0.1, *p < 0.05, **p < 0.01, ****p < 0.0001.

The first question was whether any information transferred between real and virtual environments within the maze-like environment. Using the performance (average number of errors) at Test Phase 1 of both the negative control groups (NoPre-V and NoPre-R) and the experimental groups (V-R and R-V), we performed a 2 × 2 ANOVA across the presence of a pre-exposure (with and without pre-exposure) and testing condition (virtual and real). In Test Phase 1, we found a significant main effect of pre-exposure (Figure 6A), *F*_(1, 72)_ = 34.5, *p* < 0.0001, *η*^2^ = 0.32, but no main effect of testing condition, *F*_(1, 72)_ = 0.01, *p* = 0.9, *η*^2^ = 0.00, or interaction, *F*_(1, 72)_ = 1.51, *p* = 0.22, *η*^2^ = 0.01. *Post-hoc* analyses (Sidak’s correction for multiple comparisons) revealed that both experimental groups, V-R (M = 3.48, SD = 2.26) and R-V (M = 3.02, SD = 2.09), were significantly different than both negative controls, NoPre-V (M = 5.93, SD = 1.64) and NoPre-R (M = 5.38, SD = 1.02), suggesting that general spatial information transferred between real and virtual experiences, even in a task requiring the navigation of a maze.

#### What Is the Extent of Spatial Information Transfer Between Congruent (V-V and R-R) and Incongruent Experiences (V-R and R-V) of the OLM?

To determine how well-information transferred between real and virtual experiences, we performed a 2 × 2 ANOVA across pre-exposure condition (virtual and real) and test conditions (virtual and real) to compare our experimental groups (V-R and R-V) and our positive controls (V-V and R-R). In Test Phase 1, we found no main effect of pre-exposure (Figure 6B), *F*_(1, 47)_ = 0.62, *p* = 0.43, *η*^2^ = 0.01, testing condition, *F*_(1, 47)_ = 0.00005, *p* = 0.99, *η*^2^ = 0.00, or interaction, *F*_(1, 47)_ = 1.01, *p* = 0.32, *η*^2^ = 0.02. In order to better understand these null effects, we ran a Bayesian analysis and found that the null model was the best predictor of the data with all other models being less likely (pre-exposure condition, BF_10_ = 0.33, test condition, BF_10_ = 0.28, interaction, BF_10_ = 0.09, and test condition + pre-exposure + interaction, BF_10_ = 0.05). These data suggest that spatial information transferred just as well between incongruent (V-R and R-V) and congruent experiences (V-V and R-R).

#### How Flexible Is the Transfer of Information When Starting From a Novel Location?

As we were not able to simulate the no pre-exposure conditions for Test Phase 2, we could not investigate the NoPre-V and NoPre-R conditions. To determine how flexibly information transferred between real and virtual experiences when tested from a novel location, we performed a 2 × 2 ANOVA across pre-exposure condition (virtual and real) and test conditions (virtual and real). We found no significant main effects of pre-exposure, *F*_(1, 47)_ = 1.04, *p* = 0.31, *η*^2^ = 0.03, test condition, *F*_(1, 47)_ = 0.53, *p* = 0.46, *η*^2^ = 0.01, or interaction, *F*_(1, 47)_ = 0.07, *p* = 0.79, *η*^2^ = 0.002. In order to better understand these null effects, we ran a Bayesian analysis and found that the null model was the best predictor of the data with all other models being less likely (pre-exposure condition, BF_10_ = 0.43, test condition, BF_10_ = 0.36, interaction, BF_10_ = 0.18 and test condition + pre-exposure + interaction, BF_10_ = 0.06). These data suggest that even from a novel location, spatial information transferred just as well between incongruent experiences (V-R and R-V) and congruent experiences (V-V and R-R).

#### Do the Types of Navigation Errors Participants Make Depend on the Testing Condition?

An advantage of using a maze environment is that we could begin to investigate how participants navigated and the types of navigation strategies used. Given the vaguely similar T-maze is used to distinguish place from response learners (see Experiment 3), we looked at a similar analysis in the OLM. The OLM was designed to be isomorphic, with two start locations at opposing sides of the maze (Figure 5A). In addition, all objects had a paired counterpart that existed in the same relative location to either Start Position 1 or Start Position 2 (see Materials and Methods). In Test Phase 2, when participants started from the alternate start location, we identified the types of errors participants made based on the location of the error. For example, if the target object in Test Phase 2 was #7 (gray cone), any errors on the opposite side of the maze (#3, #4, #2, #1) were considered a response error. Errors on the same side of the maze (#5, #6, #8) were considered place errors. Once we classified the types of errors made for each object in Phase 3, we calculated a simple index score [(place errors—response errors)/total errors] to determine if participants showed a stronger place bias or a response bias. We performed a 2 × 2 ANOVA across pre-exposure condition (virtual and real) and test condition (virtual and real) and found a significant main effect of test condition (Figure 6C), *F*_(1, 47)_ = 17.63, *p* < 0.0001, *η*^2^ = 0.27, but not pre-exposure condition, *F*_(1, 47)_ = 0.14, *p* = 0.71, *η*^2^ = 0.002, and no significant interaction, *F*_(1, 47)_ = 0.36, *p* = 0.54, *η*^2^ = 0.006. *Post-hoc* analyses (Sidak’s corrected for multiple comparisons) revealed a significant difference in the types of errors made with those tested in the Real-OLM maze, V-R (M = 0.41, SD = 0.42) and R-R (M = 0.29, SD = 0.51), making more place-based errors and those tested in the Virtual-OLM, V-V (M = −0.19, SD = 0.36) and R-V (M = −0.17, SD = 0.50), making more response-based errors.

#### Was Information About the Spatial Location of the Objects or the Layout of the Maze or Both Necessary for Learning the Maze?

A second advantage of using a maze environment is that we could begin to assess the type of information that might transfer between experiences. Given the time constraints of using the Real-OLM, we were only able to address this question in the virtual environment. The maze only (M-V) group was pre-exposed to the virtual maze by itself (with no objects) and tested in the full Virtual-OLM with objects. The objects only (O-V) group was pre-exposed to the objects in the virtual experience, by themselves (in the correct spatial locations) but no exposure to the maze, and then tested in the full Virtual-OLM. Comparing across all five groups that were tested in the Virtual-OLM (Figure 4A; NoPre-V, V-V, R-V, M-V, O-V) at Test Phase 1, we performed a one-way ANOVA and found a significant difference across groups (Figure 6D), *F*_(4, 78)_ = 13.12, *p* < 0.0001, *η*^2^ = 0.4. *Post-hoc* analyses (Sidak’s correction for multiple comparisons) revealed that the negative control, NoPre-V (M = 5.83, SD = 1.60), was significantly different than all groups (V-V: M = 2.90, SD = 2.00; R-V: M = 3.02, SD = 2.10; MV: M = 3.52, SD = 1.24; OV: M = 2.05, SD = 2.06), with no other comparisons being significant. These data suggested that information learned during a pre-exposure of either the object only (O-V) or maze only (M-V) conditions, aided in their ability to perform in the Virtual-OLM at test.

## EXPERIMENT 3: T-MAZE

While Experiments 1 and 2 clearly demonstrated that general spatial information could transfer between real and virtual environments, several of our findings suggested that the information may not have transferred equally between the two conditions. During Test Phase 2 of the OLT (Experiment 1), the negative control NoPre-R group learned from Test Phase 1, such that they performed better in Test Phase 2, whereas the negative control NoPre-V group did not (Figure 3C). In the OLM (Experiment 2), groups tested in the Virtual-OLM tended to make more response-based errors compared to the groups tested in the Real-OLM (Figure 6C). These results suggest that while there may not have been a difference in overall performance, the strategies people used to navigate or explore the two environments may be different. One obvious difference between the real and virtual tasks used in our experiments is the fact that all virtual tasks were performed sitting at a computer. We hypothesized that without the perceptual and vestibular cues that accompany navigation in the real world (Chance et al., 1998; Richardson et al., 1999; Hegarty et al., 2006; Waller and Greenauer, 2007; Hejtmanek et al., 2020), people were biased toward a response-based strategy because they were performing the task on a flat, 2D computer screen. To test this hypothesis, we created a virtual T-maze task and used a virtual reality headset to investigate the effects of proprioception on navigation in a virtual environment.

**FIGURE 7 |:**
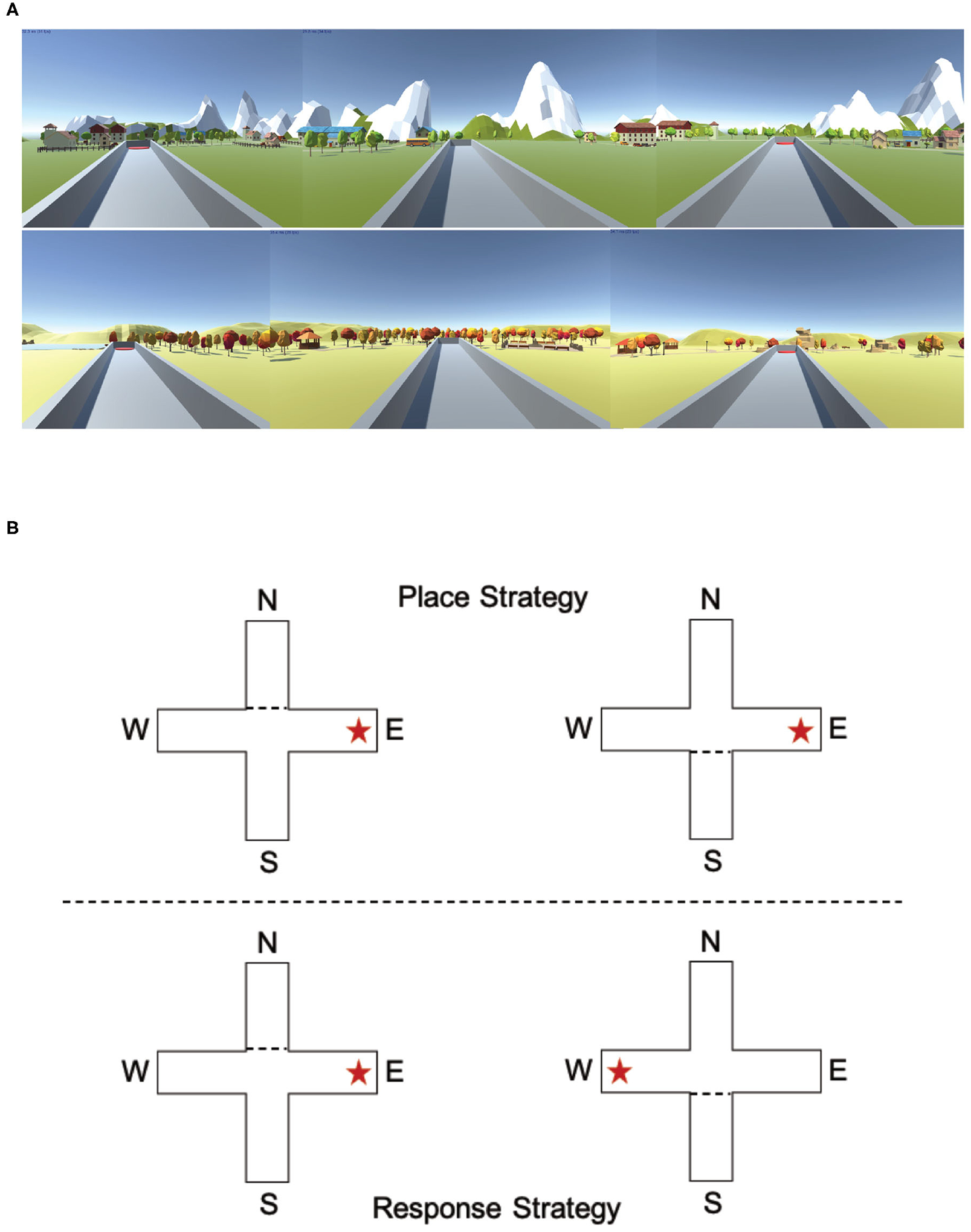
Experiencing the T-maze through either a desktop computer or a virtual reality headset could impact navigation strategy. **(A)** Example images of the two different environments used in the computer version of the T-maze. **(B)** Example descriptions of a place strategy and a response strategy. In the place condition (example image above), the target arm is always the East platform, regardless of start position (North or South). In the response condition (example image above), the target arm is always on the right arm, regardless of start position (North or South).

The T-maze task has been used in animal models to determine an animal’s preferred navigation strategy (Packard and McGaugh, 1996; Tomás Pereira et al., 2015): place or response (Figure 7B). A place strategy requires an understanding of how a target location relates to the environment (east, west, north, south, etc.) and is thought to rely on the hippocampus. A response strategy requires an understanding of how a target location relates to oneself (turn left or turn right) and is thought to be dependent on the striatum. Spontaneous navigation strategy has previously been shown to correlate with both gray matter density and activity within the hippocampus (place learners) and the caudate nucleus (response learners) (Bohbot et al., 2007).

### MATERIALS AND METHODS

#### Participants

For the computer T-maze there were 77 participants (39 female, 38 male; Mean age: 20.62 years, SD: 1.83) and for the virtual reality T-maze there were 50 participants (23 female, 27 male; Mean age: 20.28 years, SD: 1.58). All participants were recruited through the UCI Sona Systems and signed consent forms approved and conducted in compliance with the Institutional Review Board of the University of California at Irvine.

#### T-Maze

The T-maze task was designed and created using Unity (www.unity.com; Technologies Unity, 2005) and consisted of a gray start box, an elevated plus maze and two different environments (Figure 7A; Spring and Fall) that contained mountains, trees, rock formations, and small town features. The elevated plus maze consisted of four arms (North, South, East, and West) with four platforms (two start platforms and two potential target platforms) at the end of each arm (Figure 7B). There were two start locations (North and South) and two possible target locations (East and West).

We followed a T-maze procedure similar to those used in animal studies (Tomás Pereira et al., 2015). There were two versions of the T-maze (Figure 7B): one in which the participants were forced to use a place strategy to complete the task and one in which participants were forced to use a response strategy to complete the task. In the place strategy version, the target platform was always in a consistent place, regardless of the start location (always the East or West platform). In the response strategy version, the target platform was dependent on the start positions (always the left or right platform). When participants started at the North location, the South arm was blocked with a box preventing participants from entering the arm. When participants started at the South location, the North arm was blocked with a box preventing participants from entering the arm. In total, all participants performed four blocks of T-maze tasks (two place and two response T-maze tasks, randomized, presented in alternating order, and counter balanced), with counterbalanced presentations of the environments and randomization of both start and target locations. There was no discernable difference between the place T-maze block and the response T-maze block. At the end of each block, the participants were allowed to have a 5-min break to stand, stretch, and drink water.

Starting each trial in the gray start box, participants walked to a visible teleporter on the ground that randomly teleported them to one of the two start locations on either the North or South side of the elevated plus maze. Participants then proceeded to the center of the T maze and made a choice of the left or right platforms (response) or East or West platforms (place). Once the participants made a choice, they proceeded down the arm, stood on the platform, and the computer informed them if they made the correct choice by displaying “You found the platform!” in the center of the screen. Once the correct choice was made, the participant was given 5 s before being teleported back to the gray start box to continue on to the next trial. If the participant made an incorrect choice, the participant had to proceed to the other arm and stand on the correct platform. Through trial and error, the participants had to determine the correct strategy (response or place) to complete the task. The start location remained the same until the participant successfully found the platform on two consecutive trials with no mistakes. Upon two correct consecutive trials, the start location switched. In order to complete the block, the participants had to correctly pick the target platform six times in a row with no mistakes. The T-maze program recorded both the time and number of trials and errors for all runs.

#### Virtual Reality T-Maze

While one experimental group performed the T-maze task on iMac computers, the other experimental group performed the exact same T-maze task program using a virtual reality headset and physically walked in space. For the virtual reality headset, we used an HTC VIVE Pro (www.vive.com; HTC, 2016) and virtual reality sensors were placed at the corners of a 10′ × 10′ space, allowing us plenty of space for participants to navigate in the virtual T-maze. Participants were given 1 min to look and walk around the gray start box in order to acclimate to the virtual reality experience. Two experimenters were present at all times, one ran the task and the other made sure the cable connecting the headset did not tangle or interfere with the participant.

#### Spontaneous Navigation Strategy

In this T-maze paradigm, the starting location changed to the opposite side after two correct trials. We determined the participant’s spontaneous strategy by identifying the first-choice participants made after the switch to the new starting location. Based on this choice, participants were classified as either “default-placers” if they made a place-based choice, or “default-responsers” if they made a response-based choice. For example, if a participant started at the South start location with the East/right platform being the target, after finding the correct platform twice, they switched to the north start location. If the participant decided to go “West/right,” they were classified as a “default-responser” and if the participant decided to go “East/left” they were classified as a “default-placer.”

### RESULTS—EXPERIMENT 3

#### Did Navigation Strategy on the T-Maze Differ Based on Task Delivery via a Computer Screen vs. a Virtual Reality Headset?

**FIGURE 8 |:**
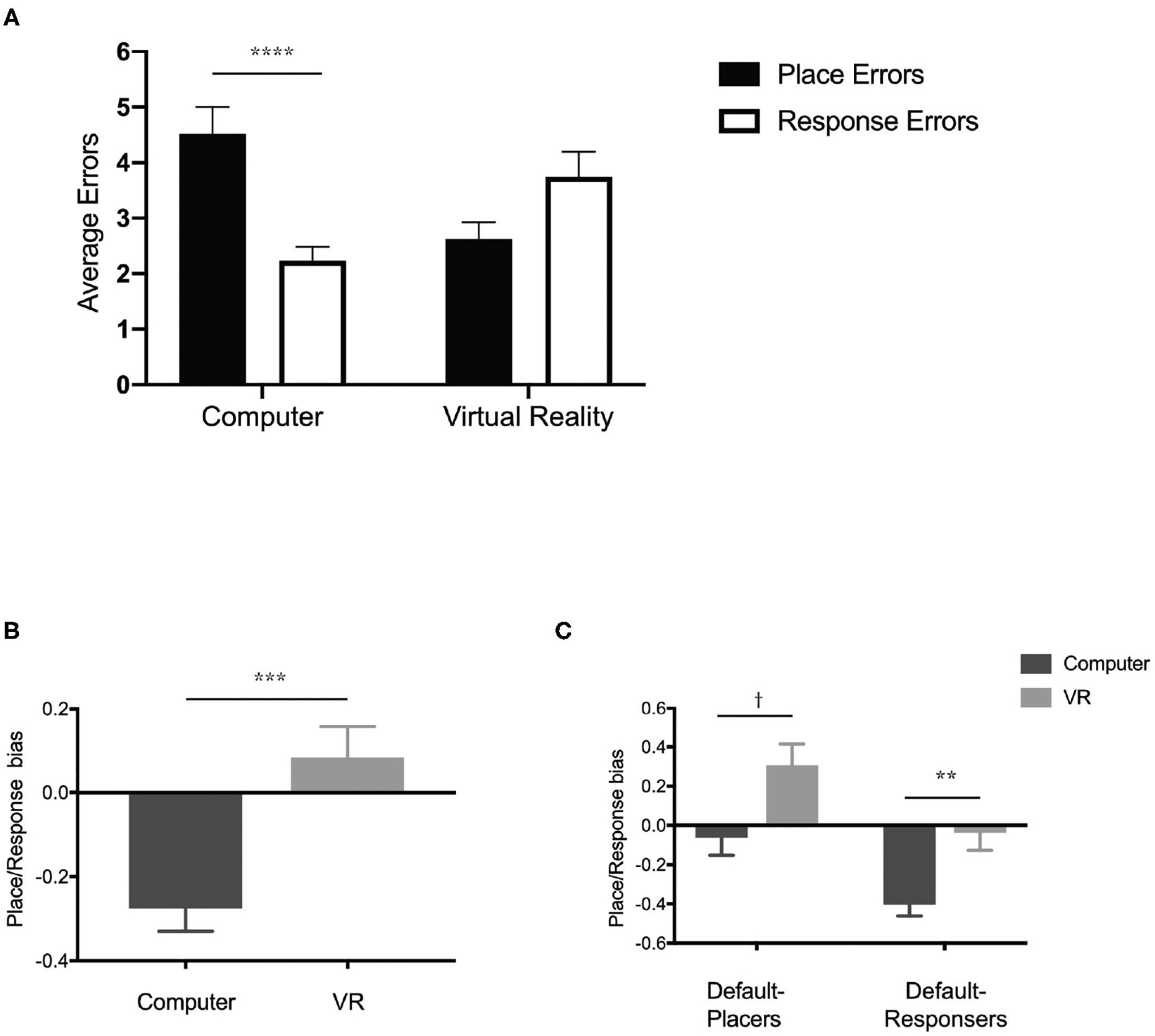
Experiencing the T-maze through either a desktop computer or a virtual reality headset could impact navigation strategy. **(A)** Performance (average errors in either the place condition or response condition) on the T-maze when performed on either a desktop computer or using a virtual reality headset. **(B)** Navigation strategy biases (<0 is more response biased and >0 is more place biased) of groups tested on a desktop computer or a virtual reality headset. **(C)** Navigation strategy biases (<0 is more response biased and >0 is more place biased) based on condition (Computer and virtual reality) and initial navigation strategy (Default-Placers and Default-Responsers). All data are presented as mean ± SEM, *^†^p* < 0.1, **p < 0.01, ***p < 0.001, ****p < 0.0001.

To determine whether the proprioceptive cues provided by navigating the physical world with a virtual reality headset influenced participants’ navigation strategy, we performed a 2 × 2 ANOVA (Figure 8A) comparing average errors across condition (computer and virtual reality) and the type of navigation error (place errors and response errors). We found a significant interaction [*F*_(1, 125)_ = 16.19, *p* < 0.0001, *η*^2^ = 0.07] but no significant main effect of condition [*F*_(1, 125)_ = 0.26, *p* = 0.61, *η*^2^ = 0.002) or type of navigation error [*F*_(1, 125)_ = 1.89, *p* = 0.17, *η*^2^ = 0.01). A *post-hoc* analysis (Sidak’s correction for multiple comparisons) revealed a significant difference between the type of navigation error in the computer condition (*p* < 0.0001) but not the virtual reality condition. While the comparison of navigation error type within the virtual reality condition did not survive multiple comparisons, there was a trend toward a difference (corrected: *p* = 0.09). These data suggest that when performing the T-maze task on a computer screen, we found that participants made significantly fewer errors in the response condition than the place condition. When performing the same T-maze task using a virtual reality headset, there was no longer a statistical difference between response and place conditions. In fact, participants’ strategy preference in the virtual reality condition seemed to have flipped from response to place. Participants made fewer errors in the place condition compared to the response condition. Using a simple index score [(place errors—response errors)/total errors], we calculated a place/response bias score (response bias <0 and place bias >0) for participants who performed the T-maze in either the computer condition (M = −0.27, SD = 0.49) or the virtual reality headset condition (M = 0.08, SD = 0.53), finding a significant difference in strategy preference between groups (Figure 8B) unpaired *t*-test; *t*_(125)_ = 3.88, *p* < 0.001, Cohen’s *d* = 0.67. Together, these data suggested that simply modifying the experience (computer or virtual reality headset) of the user could dictate their navigation strategy as assessed by a virtual T-maze task.

#### Is Spontaneous Navigation Strategy Influenced by Delivery via a Computer Screen vs. a Virtual Reality Headset?

Humans often adopt a spontaneous navigation strategy (or a default preference for one over the other) that correlates with differential activity in the hippocampus or caudate nucleus (Iaria et al., 2003), but it is unclear if this preferred navigation strategy is different on a computer screen vs. a virtual reality headset. To determine each participant’s spontaneous navigation strategy, we identified their initial choice on the first starting point switch (see Materials and Methods) and classified them as either “default-placers” or “default-responsers.” Regardless of condition, default-placers made up roughly 37% of participants (29 participants in the computer condition and 18 participants in the virtual reality condition) and default-responsers made up roughly 63% of participants (48 participants in the computer condition and 32 participants in the virtual reality condition), similar to previous reports (Iaria et al., 2003). A 2 × 2 ANOVA comparing response/place bias across both condition (computer and virtual reality) and spontaneous navigation group (default-placers and default-responsers) revealed a significant main effect of both condition (Figure 8C), *F*_(1, 123)_ = 13.54, *p* < 0.001, *η*^2^ = 0.01, and group, *F*_(1, 123)_ = 15.67, *p* = 0.0001, *η*^2^ = 0.1, but no interaction, *F*_(1, 123)_ = 0.0005, *p* = 0.98, *η*^2^ = 0.00. Across group (default-placers and default-responsers), default-placers were more place strategy biased and default-responsers were more response strategy biased. Across condition (computer and virtual reality), the computer condition were more response biased and the virtual reality condition were more place biased. *Post-hoc* analyses (Sidak’s correction for multiple comparisons) revealed that within the computer condition and the virtual reality condition, default-placers, and default-responsers were significantly different from one another. Default-responsers within the computer condition (M = −0.40, SD = 0.44) were more biased toward a response strategy and in the virtual reality condition (M = −0.03, SD = 0.54) did not appear to have a strong bias of place or response strategy. Default-placers within the computer condition (M = −0.06, SD = 0.49) did not have a strong bias toward place or response strategy, however in the virtual reality condition (M = 0.30, SD = 0.46) they displayed a stronger place strategy bias. These data suggest that default-placers and default-responsers are differentially influenced by testing condition in the T-maze. The virtual reality condition led to a stronger place bias only in the default-placers whereas the computer condition led to a strong response bias only in the default-responsers.

#### Are There Gender Differences in Either Spatial Memory Performance or Navigation Strategy Across Experiments?

While gender differences was not a primary goal of these studies, both the animal and human literature have suggested that gender differences are particularly prevalent in the spatial domain (Yuan et al., 2019). In total, across three separate experiments, we recruited 285 participants, of which 143 were female and 142 were male. In Experiment 1, we performed a 2 × 2 ANOVA comparing average error rates of Test Phase 1 across both group (NoPre-V, NoPre-R, R-V, V-R, V-V, R-R) and gender (female and male) and found a significant main effect of group [*F*_(5, 65)_ = 9.61, *p* < 0.0001, *η*^2^ = 0.41] but no main effect of gender [*F*_(1, 65)_ = 0.005, *p* = 0.95, *η*^2^ = 0.00] or interaction [*F*_(5, 65)_ = 0.63, *p* = 0.68, *η*^2^ = 0.03). In addition, we also performed a 2 × 2 ANOVA comparing average error rates of Test Phase 2 across both group (NoPre-V, NoPre-R, R-V, V-R, V-V, R-R) and gender (female and male) and found a significant main effect of group [*F*_(5, 65)_ = 9.87, *p* < 0.0001, *η*^2^ = 0.42] but no main effect of gender [*F*_(1, 65)_ = 1.80, *p* = 0.18, *η*^2^ = 0.01] or interaction [*F*_(5, 65)_ = 0.41, *p* = 0.84, *η*^2^ = 0.02)].

In Experiment 2, we performed a 2 × 2 ANOVA comparing average error rates of Test Phase 1 across both group (NoPre-V, NoPre-R, R-V, V-R, V-V, R-R) and gender (female and male) and found a significant main effect of group [*F*_(5, 87)_ = 12.19, *p* < 0.0001, *η*^2^ = 0.4) but no main effect of gender [*F*_(1, 87)_ = 0.47, *p* = 0.50, *η*^2^ = 0.003) or interaction [*F*_(5, 87)_ = 0.56, *p* = 0.72, *η*^2^ = 0.2). In addition, we also performed a 2 × 2 ANOVA comparing average error rates of Test Phase 2 across both group (R-V, V-R, V-V, R-R) and gender (female and male) and did not find a significant main effect of group [*F*_(3, 43)_ = 0.35, *p* = 0.78, *η*^2^ = 0.02), gender [*F*_(1, 43)_ = 1.10, *p* = 0.30, *η*^2^ = 0.02), or interaction (*F*_(3, 43)_ = 0.86, *p* = 0.47, *η*^2^ = 0.05].

In Experiment 3, we performed a 2 × 2 ANOVA comparing average error rates of the computer condition across navigation strategy error (place and response) and gender (female and male). We found a significant main effect of navigation strategy error [*F*_(1, 75)_ = 17.79, *p* < 0.0001, *η*^2^ = 0.1], but no main effect of gender [*F*_(1, 75)_ = 0.13, *p* = 0.72, *η*^2^ = 0.002] or interaction [*F*_(1, 75)_ = 0.75, *p* = 0.39, *η*^2^ = 0.004]. In addition, we performed a 2 × 2 ANOVA comparing average error rates of the virtual reality condition across navigation strategy error (place and response) and gender (female and male). We did not find a significant main effect of navigation strategy error [*F*_(1, 48)_ = 3.39, *p* = 0.07, *η*^2^ = 0.04], gender [*F*_(1, 48)_ = 0.49, *p* = 0.49, *η*^2^ = 0.01] or interaction [*F*_(1 48)_ = 0.71, *p* = 0.40, *η*^2^ = 0.01].

Therefore, across all three experiments, which consisted of both real world and virtual world tests of spatial ability, we did not find any evidence for gender differences in either spatial memory performance or navigation strategy.

## DISCUSSION

Here, we presented three different experiments in which we investigated the transfer of spatial information between real and virtual environments. In Experiment 1, we found that general spatial knowledge about object location transferred between real and virtual environments. Pre-exposure and testing in opposing conditions (virtual → real or real → virtual) led to similar performances as pre-exposure and testing in the same conditions (real → real or virtual → virtual). In Experiment 2, we again showed that spatial information transferred between real and virtual environments during the navigation of a maze-like environment. Furthermore, spatial knowledge about object location and maze layout transferred within the virtual environment, demonstrated when pre-exposing participants to either the object locations only (without the maze) or the layout of the maze only (without the objects) led to improved performance compared to no pre-exposure at all. Lastly, we showed that while general spatial knowledge transferred between real and virtual environments, the way in which individuals explored or navigated these environments was influenced by the experimental platform. Navigation strategy (place or response) varied on a T-maze task depending on whether it was performed on a computer screen or using a virtual reality headset.

The results of experiments 1 and 2 are consistent with a recent study which also explored the transfer of spatial knowledge between virtual world and real world environments (Hejtmanek et al., 2020). In this study, participants learned the layout of a campus building through real world navigation, immersive VR navigation using a head mounted display and omnidirectional treadmill, or desktop VR navigation. Participants were then tested on their transfer of knowledge by navigating the campus building in the real world. While real world navigation led to the best performance, both virtual conditions (immersive VR and desktop VR) demonstrated transfer to the real world with immersive VR providing some advantages over desktop VR.

In our study, experiments 1 and 2 demonstrated that spatial information could reliably transfer between real and virtual environments when the virtual environment was modeled after the real environment. Regardless of the pre-exposure experience, spatial knowledge about the objects’ locations transferred, such that performance in the experimental conditions (V-R and R-V) was equivalent to the positive controls (V-V and R-R) and significantly better than the negative controls (NoPre-V and NoPre-R). However, there were two results from Experiments 1 and 2 that suggested spatial information did not transfer equally, or to the same extent, between experiences. In Experiment 1, we expected to observe learning between Test Phase 1 and Test Phase 2 in our negative control groups (NoPre-V and NoPre-R). Even though these groups did not receive a pre-exposure, we expected them to demonstrate learning at Test Phase 2 if Test Phase 1 provided a pre-exposure event for these groups. However, learning occurred in the group exposed to the real condition (NoPre-R) but not the virtual condition (NoPre-V) indicating a specific impairment in the ability to learn spatial locations in a virtual environment from a novel starting point (Test Phase 2). Importantly, the addition of a pre-exposure (V-V) rescued this impairment in learning. In Experiment 2, we analyzed the types of errors participants made and found that groups tested in the virtual environment (R-V and V-V) had a higher percentage of response-based errors whereas groups tested in the real environment (V-R and R-R) had a higher percentage of place-based errors. In both Experiment 1 and 2, groups were tested on their spatial knowledge from a novel location. Similar to a probe trial in the water maze (Morris, 1981) or the ability to use shortcuts (Maguire et al., 1998), being able to navigate from a novel location is an indicator of an allocentric or map-based representation of space. These results suggest that while there was no difference in the overall performance of these tasks, subtle differences may lie in whether these two experiences promote the formation of a cognitive map that may be dependent on hippocampal involvement.

To test the influence of proprioception on navigation strategy, we employed a virtual version of the T-maze task, a commonly used navigation task designed to differentiate between response and place strategies. We found that navigation strategy was dependent on the platform participants used to experience the task. Participants demonstrated a strong preference for a response-based strategy when performing the task on a computer screen and strong preference for a place-based strategy when performing the task using a virtual reality headset. The behavioral task was identical between conditions (task protocol, visual design of the maze, and environment, etc.) with the primary difference being the experimental delivery experience. While the computer screen required a mouse and keyboard to navigate, participants in the virtual reality headset group had to move and walk around a physical space in order to complete the task. These data suggest that addition of perceptual and proprioceptive cues had a significant influence on individual navigation. In addition, the novelty of using a virtual reality headset may have promoted increased attention to the surroundings which may have caused greater reliance on place strategies, but our task design did not allow us to explore that possibility further.

The role of proprioception was a clear difference between the real and virtual experiences of Experiments 1 and 2, and has been shown to impact performance in spatial tasks (Chance et al., 1998; Richardson et al., 1999). In the real versions of both tasks, participants walked around the real world in the Pre-Exposure Phase and both Test Phases whereas in the virtual versions, participants navigated around using the keyboard and mouse. We have some evidence that proprioception influenced navigation, observing differences in the types of navigation errors (response vs. place) between real and virtual environments in Experiments 2 and 3. However, across both Experiment 1 and 2, proprioception did not appear to have a significant impact on the transfer of spatial knowledge, specifically about object location, between environments. Importantly, our primary metric used across these experiments was based on errors in order to make direct comparisons between the real and virtual experiences. Other metrics, such as travel time, may better reflect the influence of proprioception. These data suggest that while information about object-location transferred well between real and virtual environments, the processes underlying this learning may depend on experience and the proprioceptive feedback from the environment. While virtual environments have clear value as they allow us to probe the underlying neural mechanisms of spatial cognition, they have their limits. Fortunately, these limitations can be addressed with the use of virtual reality technology.

A limitation of the current study is the relatively modest sample size across each of the groups, especially when split by gender. There have been numerous reports of robust differences between men and women in regards to spatial strategies or cognition, having been observed in both real (Malinowski and Gillespie, 2001; Vashro and Cashdan, 2015) and virtual (Astur et al., 1998; Driscoll et al., 2005; Choi et al., 2006; Sneider et al., 2015; Padilla et al., 2017) environments. However, across all three experiments, in both real and virtual environments, we did not observe any evidence for reliable effects of gender on spatial learning, memory, or navigation. There are a several factors that could contribute to potential gender differences in spatial cognition, including size and scale of the environments (Padilla et al., 2017), episodic memory (Sargent et al., 2019), spatial cues (Livingstone-Lee et al., 2011), hormones (Driscoll et al., 2005), as well as social and cultural influences (Hoffman et al., 2011; Vashro and Cashdan, 2015). In addition, gender differences within the spatial domain have been shown to be task specific and can be influenced by procedure (Voyer et al., 1995). Any interpretation on the lack of gender differences in our results would be merely speculation. Importantly, the majority of studies that have observed gender differences in humans were performed in virtual environments, once again highlighting the need to further explore how virtual experiences parallel the real world.

Finally, Experiment 2 was a quasi-experiment as there were several factors that were out of our control. The life-size maze was a unique opportunity for us to investigate humans exploring a real-world environment and while the design of this experiment was preplanned, factors such as time made it difficult to execute the procedure to the same standards as Experiment 1 or Experiment 3. In addition, since these experiments were run outdoors, it was difficult to account for the influence of novelty or other aspects of real-world experiments (such as exercise or weather).

In today’s modern world, virtual experiences have become increasingly commonplace. As we travel further down this digital world, it is imperative that we understand the relationship between these virtual and real interactions so that we can create virtual experiences that engage us and offer real application to the physical world around us. The study presented here directly addresses the notion of transfer and suggests that we are able to learn spatial information about real-world locations, without any prior experience or knowledge of the location, through a virtual experience. While the conditions of these experiments were designed to promote transfer, the goal of this study was to not only demonstrate that transfer exists but to begin to understand the extent of this transfer. Spatial memory transferred well between real and virtual experiences, however, the way in which people explored and encoded this information was dictated by the proprioceptive and perceptual cues provided by the experience.

## Data Availability

The raw data supporting the conclusions of this article will be made available by the authors, without undue reservation.
